# Real-time size modulation and synchronization of a microfluidic dropmaker with pulsed surface acoustic waves (SAW)

**DOI:** 10.1038/s41598-018-22529-w

**Published:** 2018-03-14

**Authors:** Lothar Schmid, Thomas Franke

**Affiliations:** Chair of Biomedical Engineering, School of Engineering, University of Glasgow, Oakfield Avenue, G12 8LT Glasgow, United Kingdom

## Abstract

We show that a microfluidic flow focusing drop maker can be synchronized to a surface acoustic waves (SAW) triggered by an external electric signal. In this way droplet rate and volume can be controlled over a wide range of values in real time. Using SAW, the drop formation rate of a regularly operating water in oil drop maker without SAW can be increased by acoustically enforcing the drop pinch-off and thereby reducing the volume. Drop makers of square cross-sections (w = h = 30 µm, with width w and height h) that produce large drops of length l = 10 w can be triggered to produce drops as short as l ~ 2w, approaching the geometical limit l = w without changing the flow rates. Unlike devices that adjust drop size by changing the flow rates the acoustic dropmaker has very short transients allowing to adjust the size of every single drop. This allows us to produce custom made emulsions with a defined size distribution as demonstrated here not only for a monodisperse emulsion but also for binary emulsions with drops of alternating size. Moreover, we show that the robustness and monodispersity of our devices is enhanced compared to purely flow driven drop makers in the absence of acoustic synchronization.

## Introduction

Droplet based microfluidics has been shown to be a powerful tool for biological^1,2^ and synthetic systems^3,4^. Drops can serve as containers for cells and bacteria^5^ or have been used as templates for gel-like particles^6^. Formation of drops involve two immiscible phases of fluids – the continuous and the dispersed (droplet) phase – that both constitute a single emulsion. Generation of single emulsion drops have been accomplished in glass micro-capillaries^7^ providing extreme speed and throughput and polydimethylsiloxane (PDMS) channels^8–11^ that are more flexible in design and capable of being integrated in more complex device architecture. In PDMS, multiple operations such as mixing, merging, splitting and pico-injection have been realized and combined on a microfluidic chip employing a multitude of physical effects^12^. However, controlling the droplet size and volume still remains challenging. Precise adjustment of drop size is essential for many droplet based applications – for example when combining drops to accurately control and track the concentration of molecules in the drops. In a series of studies Garstecki *et al*.^13–15^ have used syringe pump driven flow in different device geometries and explained the dependence of drop size on flow rates in detail. By pressurizing air-filled side-channels Abate *et al*. have changed the effective diameter of the nozzle at which drop formation occurs yielding a range of drop sizes^16^. Also, dielectric forces have been employed to produce “sound by drops” that is based on modulation of drop sizes encoding for frequency^17^. Most recently, monodisperse emulsions have been produced by surface acoustic waves in a T-junction and cross-junction geometry^18,19^. In similar approaches, SAW was used to produce droplets on demand^20^ at rates of a few drops per second, as well as to split droplets^21^.

However, in many of these approaches transients can be very long and response times are slow. For example syringe pumps have response time up to minutes^22^ and are difficult to gauge. Here, we introduce for the first time the acoustic control of droplet size in real-time in a continuously producing drop-maker. Using acoustic pulses as short as 1.5 ms we are able to synchronize aqueous drop formation in oil with an external signal given by a programmable signal generator for a wide range of droplet rates and volumes. We show that we can acoustically shorten the droplet dimension of a drop maker approaching the limit of drop formation in the squeezing^14,15^ regime l = w without a change in externally driven flow rate. Our flow focusing drop maker with a square cross section of 30 µm is able to produce drops as short as 63 µm. The acousto-fluidic device enables us to provide drops with sizes on demand and at high monodispersity and rate.

## Results

The dropmaker regularly produces droplets without application of the SAW. Drop size and rate in absence of SAW modulation depend on the flow rates and channel geometry, with droplet length *l* normalized to the channel width at the junction *w* in the range of 6.5 < *l*/*w* < 13.3.

**Figure 1:**
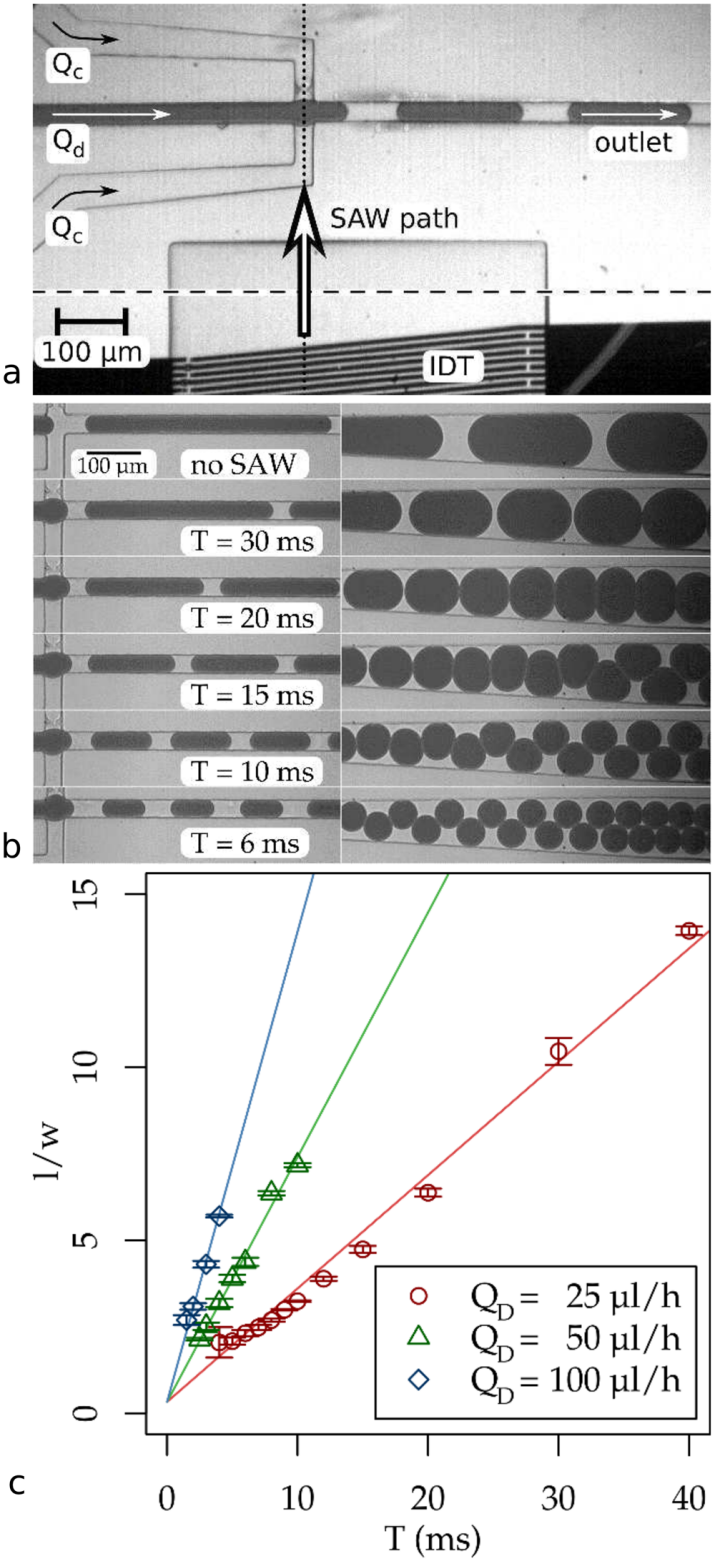
Setup and drop length modulation. **(a)** Top view of the dropmaker with IDT. **(b)** Droplets in the outlet channel near the junction and further downstream without SAW (topmost) and with SAW at different modulation periods *T* and a pulse length of *τ* = 2.8 ms. A video showing droplet generation with and without SAW at the junction is available as supplementary video S1. **(c)** Droplet length (normalized to channel width w at the junction) vs. SAW pulse periodicity *T*. The droplet volume increases linearly with *T*, with the slope corresponding to the flow rate of the dispersed liquid *Q*_*d*_.

We then apply a rectangular SAW pulse of varying period *T* and constant length. In this regime the SAW pulse pinches off the drops from the aqueous phase and the dropmaker continuously produces monodisperse drops at a rate that is perfectly synchronized to the external modulation signal. For all flow rates of the dispersed phase *Q*_*d*_ the normalized droplet length *l*/*w* follows a linear relation with the period *T* as shown in Fig. 1(c). The slope of the curve is a function of *Q*_*d*_ and can by estimated from a simple model: describing the drop shape by a cylinder with two spherical caps in the front and at the rear allows us to write the normalized length depending on the droplet volume *V* in the following way: $l/w=V\,\ast \,4/\pi {w}^{3}+1/3$. The ratio of droplet volume V(*l*/*w)* and period *T* is the experimentally observed flow rate of the dispersed phase and almost identical to the applied flow rate of the dispersed fluid *Q*_*d*_ for all three flow rated used: 25.0 ± 0.5 µlh^−1^, 54.0 ± 1.0 µlh^−1^ and 103 ± 2.7 µlh^−1^. This demonstrates that the pulsed SAW does not change the overall flow rate of the dispersed phase *Q*_*d*_, which is set by the syringe pump. Instead, the SAW controls the timing of the pinch-off of the individual droplets. Therefore, the droplet volume is exactly the amount of dispersed fluid passing the junction during one period *T*.

As shown in Fig. 1(c), the droplets produced at given *T* by the pulsed SAW have a highly uniform size distribution: The standard deviation, normalized to droplet size, is ${\rm{\Delta }}l/l=2.4$%, with the exception of one outlier with a standard deviation of 21%. This outlier was measured at a very small droplet size $l/w=2.0$ where the dropmaker is on the verge of becoming unstable. The monodispersity using SAW in our experiments is therefore slightly higher than typical values reported in literature, where the standard deviation of droplet volume is in the range of ${\rm{\Delta }}V/V=4$% or even more^22^.

**Figure 2:**
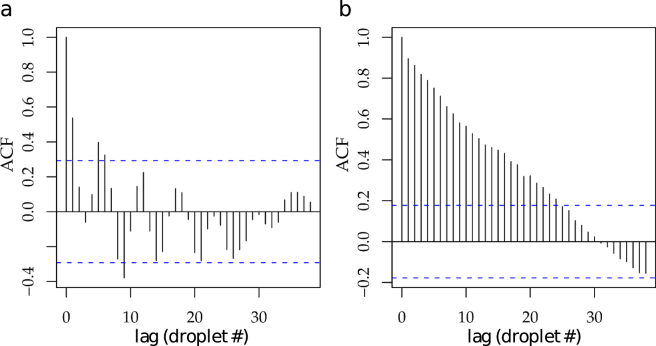
Autocorrelation of droplet length. The autocorrelation function (ACF) shows the length correlation of subsequent droplets (indicated by droplet number #) for *T* = 8 ms **(a)** before and **(b)** after switching on the pulsed SAW. Without SAW, the dropmaker exhibits periodic length oscillations that are suppressed as soon as the SAW is switched on. The decline of the ACF that occurs in the absence of SAW and the presence of SAW modulation is caused by slow drift of the droplet sizes and likely caused by drift in the syringe pump flow (see supplemental material).The blue dashed line represents the threshold for confidence level 0.95.

There is another interesting and useful effect when drop formation is forced by pulsed SAW. When producing unmodulated drops without SAW we found that drop formation is slightly unstable exhibiting length oscillations, as shown in Fig. 2(a). These oscillations are caused by the interaction of the drop formation process with droplets, that have already been formed and that are flowing further downstream. Depending on the number of drops in the section after the cross-junction a slight change of the hydrodynamic resistance in the outlet channel is generated. Hence, when a droplet flows from the thin microfluidic outlet channel into the reservoir it causes a pressure variation that can interfere with the droplet production. This causes a correlation of the size of the newly formed droplet with the one flowing into the outlet reservoir, as shown by the autocorrelation function in Fig. 2(a). However, when the SAW pulse pinches of the drop, this effect is strongly suppressed and no oscillations can be observed any more in Fig. 2(b).

**Figure 3:**
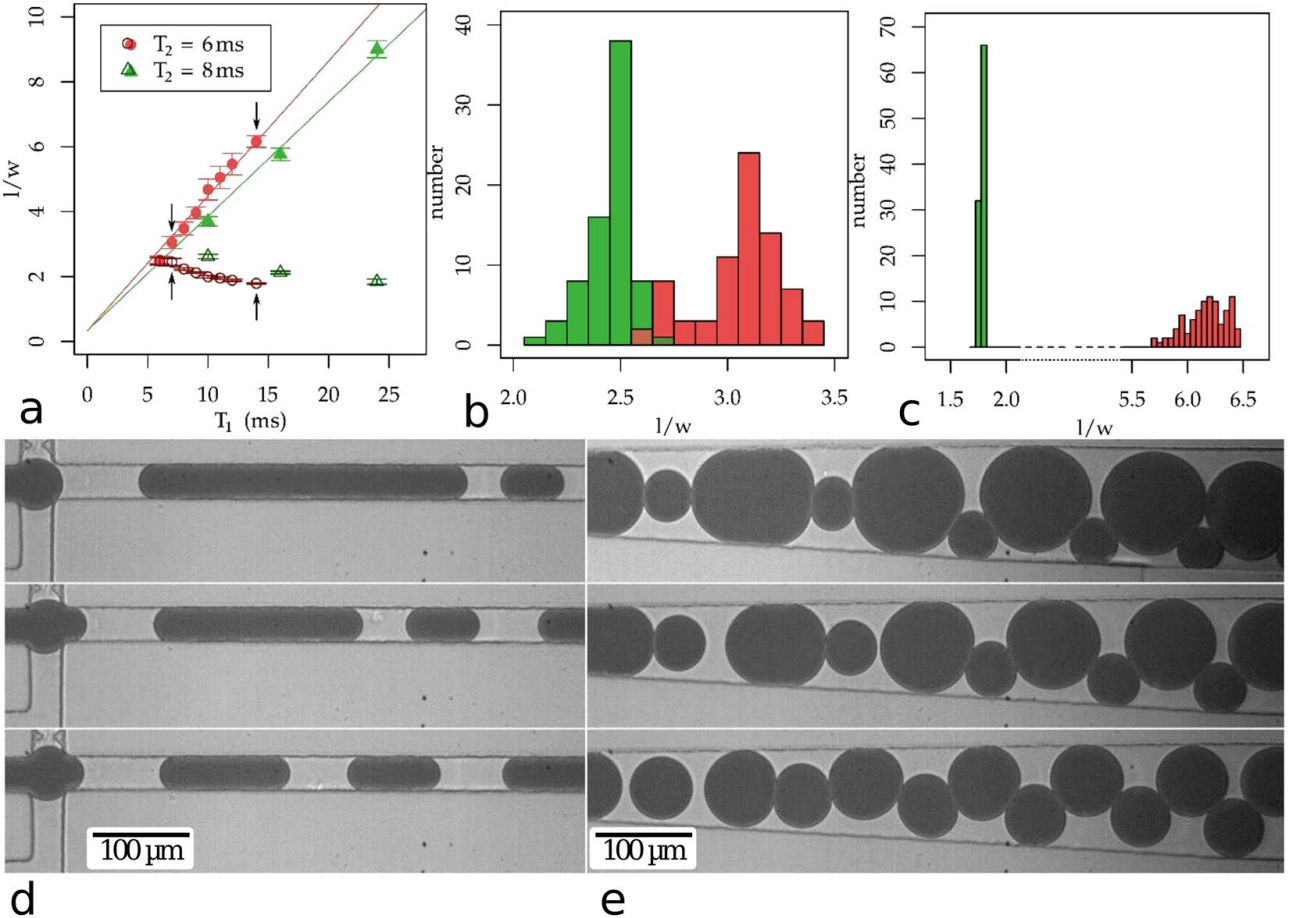
Dual pulse operation of the dropmaker. **(a)** Normalized droplet lengths at dual pulse modulation at different periodicities *T*_1_ < *T*_2_, at a flow rate *Q*_*d*_ = 25 µlh^−1^ and *T*_2_ = 6 ms and *T*_2_ = 8 ms, period *T*_1_ was varied between 6 ms <*T*_2_< 24 ms: The length of the larger droplets linearly increases with *T*_1_ (filled red circles and filled green triangles), while the length of the smaller droplets slightly decreases even though *T*_2_ = 6 ms (open red circles) and *T*_2_ = 8 ms (open green triangles) is kept constant. Error bars denote the standard deviation of the droplet size and black arrows indicate the data points for which size distributions are displayed. **(b)** Size distribution of droplets at double pulse modulation with *T*_2_ = 6 ms and *T*_1_ = 7 ms; and **(c)** *T*_2_ = 6 ms and *T*_1_ = 14 ms. **(d)** Micrograph of the dual pulse experiment at three different modulation time ratios (*T*_1_:*T*_2_ = 24:8, 16:8, 10:8 with *τ* = 4 ms) at the junction (left) and downstream where the channel widens and the droplets become circular (right). A video showing binary droplet generation is available as supplementary video S2.

The precise synchronization of droplet pinch-off with an external modulation signal enables us not only to fine-tune droplet sizes to produce a monodisperse emulsion but, beyond that, to produce droplets of different sizes on demand and in real time. To demonstrate this enormous level of control, we produce a binary emulsions with a regular sequence of alternating smaller and larger drops using dual pulse modulation, where we alternate the periods of successive pulses *T*_1_ > *T*_2_, while keeping *T*_2_ constant, as shown in Fig. 3. The size of the produced droplets changes linearly with *T*_1_ similarly to the observations made in the previous experiments using single modulation. The experimental flow rate coefficients, however, differ slightly more: m = 31.7 µlh^−1^ for *T*_2_ = 6 ms and $m=26.9$ µlh^−1^ for *T*_2_ = 8 ms, at an applied flow rate of ${Q}_{d}=25$ µlh^−1^. Conversely, the size of the small droplets produced during the timespan *T*_2_ slightly decreases with increasing *T*_1_, which explains the additional volume in the larger droplets. The mean size distribution of the *T*^2^ population is almost identical to the previous experiments, with ${\rm{\Delta }}l/l=2.5$%, while the distribution for the *T*_1_ droplets is wider: ${\rm{\Delta }}l/l=4.9$%.

## Discussion

**Figure 4:**
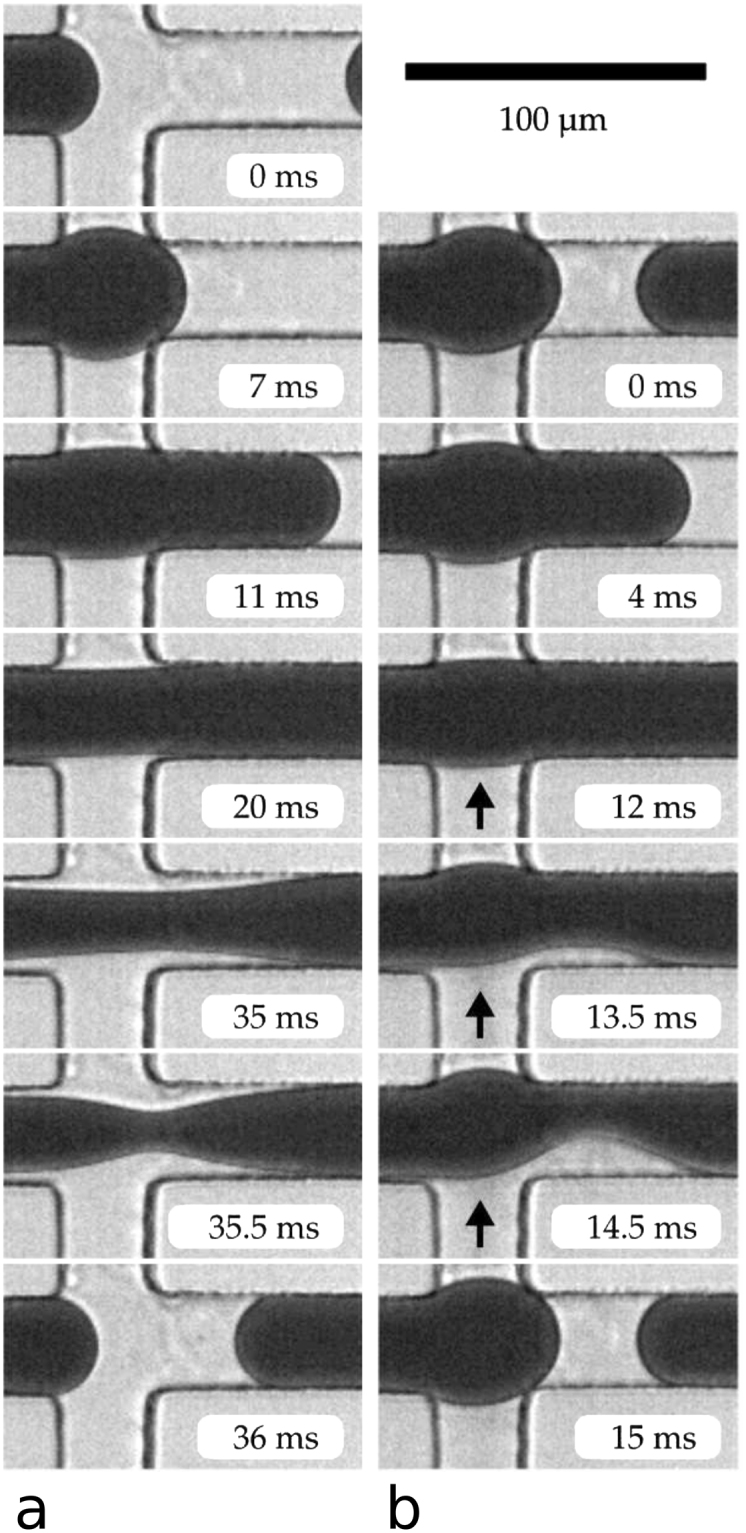
Pressure driven pinch-off mechanism. (**a**) SAW Off: without SAW pulses, gradual pressure increase in the continuous fluid upstream of the junction leads to a slow decrease of the diameter of the neck connecting the newly forming droplet to the inlet, until the neck becomes unstable (t = 35.5 ms) and the droplet pinches off, restarting the cycle. **(b)** SAW On: When the SAW is switched on at t = 12 ms (denoted by black arrows), the additional SAW pressure in the lower inlet channel leads to a rapid asymmetric pinch-off within 3 ms.

The SAW-modulation can be explained by extending a model proposed by Abate *et al*.^23,24^ for pressure-driven pinch-off of droplets in T-junction dropmakers. The cross-junction dropmaker used in our experiments operates in the “squeezing regime”^14^ and the typical process in the absence of a SAW modulation is shown in Fig. 4(a). A newly forming droplet blocks the outlet channel of the junction and causes a pressure increase in both oil inlet channels, which drives the decrease in the neck diameter connecting the newly forming droplet to the fluid in the inlet. After a critical pressure *p*_*crit*_ and neck diameter have been reached a drop spontaneously pinches off by Rayleigh instability. In case of a SAW modulation, an additional pressure is generated in the lower inlet channel due to the acoustic streaming effect, as shown by previous experimental^19^ and theoretical work^25^. This additional pressure leads to an accelerated pinch-off process since the critical pressure is attained at an earlier time, as shown in Fig. 4(b). Typical values of the critical pressure measured at similar flow conditions using Laplace-sensors^24^ (${p}_{crit}=0.6$ kPa) are in range of the SAW-generated pressures, as demonstrated in previous work^19^ and therefore further support the mechanism proposed here.

This model also accounts for some limitations of our SAW modulation device: Droplet pinch-off occurs continuously even without a SAW pulse, as in a conventional syringe pump driven cross-junction dropmaker, and cannot be stopped or further delayed by SAW modulation. Therefore, the flow rates of the dispersed *Q*_*d*_ and continuous phase *Q*_*c*_ determine an upper limit for the droplet size that cannot be further increased by the modulation. To achieve even larger drops the flow rates would have to be adjusted accordingly. The lower limit of droplet size is set by the maximum pressure the SAW can generate, as for very small droplets, a high additional SAW pressure is necessary to reach the critical value and enable the pinch-off. Furthermore, the lower limit is also narrowed by the channel dimension. We used a microchannel of 30 µm height and length. The smallest drop size for a spherical drop in the squeezing regime is therefore *l* = *w* = 30 µm. We reach drops length of *l* = 63 (l ~ 2 w) which is close to the geometric limit and the limit reported in experiments.by Garstecki^13–15^. Consequently, if the modulation frequency is too high, only every second SAW pulse pinches off a droplet. In this way higher modulation frequencies can even lead to quasiperiodic behaviour of the system.

In conclusion, we have demonstrated that pulses of SAW can synchronize drop formation in a continuously operating drop maker that allows precise control over individual drops and their size. The level of monodispersity we reach is even higher than typical values reported in the literature for other dropmakers. Since the overall flow rates of the system are not affected by the SAW actuation the impact on the microfluidic system further up- or downstream is minor, i.e. SAW modulation is compatible and does not interfere with other features integrated on the device. Moreover, we have shown that SAW can suppress pressure fluctuations associated with droplet fluidic flow and stabilizes the pressure in a microfluidic system. As a proof of the power of our approach we produced binary emulsions with two alternating sizes of drops using a dual pulse mode for the SAW. We anticipate that this method will be useful not only to produce drops of different composition than the water/HFE-oil system shown here, but also to decouple this versatile, on-demand droplet maker from other functional modules integrated on the same chip. An example of this could be the controlled formation of a monodisperse multiple emulsion in which the number and size of the inner phase drops^26,27^ or polymer micro-particles^28^ can be regulated in real time.

## Methods

For aqueous droplet production we use a hydrodynamic flow focusing geometry fabricated in a single layer PDMS (Polydimethylsiloxane) using soft lithography as described elsewhere, with a constant channel height and width at the junction of *h* = *w* = 30 μm. Briefly, the master for the PDMS mold is produced using a mask aligner to illuminated the photomask to a SU-8 photoresist (NANO^TM^ SU-8 50, MicroChem Corp.) spin coated on silicon wafers. The layer is then formed by pouring a 5 mm thick layer of PDMS onto the SU8 master and the PDMS is cured at 65 °C for 3 hours. The layer is subsequently detached from the master, inlet and outlet holes are punched with a coring tool (Harris Uni-Core Multi-Purpose Sampling Tool 0.5). The casted PDMS mold is aligned and bonded to the piezoelectric substrate made of LiNbO_3_ (Lithium niobate, 128° rot-Y-cut) by ozone-plasma treatment. The specific cut was chosen to ensure that only Rayleigh waves, i.e. transversal surface acoustic waves with a small longitudinal component are excited by the IDT and that other modes are almost completely suppressed. On the piezo-substrate a tapered interdigital transducer (TIDT) made of gold electrodes (120 fingers of 6 µm width) and an aperture of 0.5 mm) is metallized by vapor deposition and aligned in close proximity to the drop formation region as shown in Fig. 1(a). To fabricate the IDTs, layers of 10 nm Ti, 50 nm Au and 10 nm Ti were deposited onto a 17.5 × 17.5 mm piezoelectric substrate via electron beam evaporation. For mechanical protection a 200 nm thick SiO_2_ -layer was sputtered onto the LiNbO_3_-chip. The position of the SAW excited by the TIDT can be fine-tuned to precisely target the drop formation region by slightly tuning the carrier frequency, in the range of 161–171 MHz.

We continuously produce a water in oil emulsion (w/o) of aqueous drops dyed with 20 mM bromphenol blue in HFE-7500 fluorocarbon oil (3 M Novec) with 1.8% fluorosurfactant ammonium carboxylate of DuPont Krytox 157 to stabilize the droplets. The flow of the dispersed phase and the continuous phase are driven by two syringe pumps (Harvard Apparatus PHD2000 infusion) and do not change over the course of a single experiment. The flow rate of the dispersed phase *Q*_*d*_ is set to 25 µlh^−1^, 50 µlh^−1^ and 100 µlh^−1^, while the flow rate of the continuous phase is set to half of that: ${Q}_{c}={Q}_{d}/2$. The SAW is excited using a high-frequency generator (Rhode Schwarz SMP02) and the signal is amplified to a power of 800 mW using an amplifier (ZHL-2, Mini-Circuits) before fed into the interdigital transducer. The carrier frequency is pulse modulated by a rectangular modulation signal, with periods between 1.5 ms <*T* < 40 ms and pulse widths within 0.9 ms  <τ < 2.8 ms.

The assembled hybrid device is mounted on a microscope (Zeiss, Axiovert 200 M) stage and is imaged with a bright-field microscope and a high-speed camera (Fastcam 1024 PCI, Photron) using frame rates between 125 and 1000 fps.

## Electronic supplementary material

Binary droplet maker

Simulation of Autocorrelation
