# Supplementary material for: Real-time size modulation and synchronization of a microfluidic dropmaker with pulsed surface acoustic waves (SAW)
Source: Sci Rep. 2018 Mar 14;8:4541. doi: 10.1038/s41598-018-22529-w (PMC5852020; doi:10.1038/s41598-018-22529-w)
Supplement: Supplementary file 2 — Simulation of Autocorrelation [file 41598_2018_22529_MOESM2_ESM.pdf]

Supplemental Information

Real-time size modulation and synchronization of a microfluidic dropmaker with pulsed surface acoustic waves (SAW)

Lothar Schmid[a] and Thomas Franke[a]*

[a] Chair of Biomedical Engineering, School of Engineering, University of Glasgow, Oakfield Avenue, G12 8LT Glasgow, United Kingdom
* thomas.franke@glasgow.ac.uk

Remarks to the Autocorrelation and simple model for drift observed in Fig.2:

The y-axes in Fig. 2a and 2b show the autocorrelation function of the length correlation. There are two main features that we want to point out here, concerning firstly the oscillations and secondly the drift:

1.The left side of figure 2 shows the autocorrelation function of the dropmaker without SAW. There are clearly oscillations that do exist for longer times (at least 30drops as shown in the figure). These oscillations do not occur when using SAW (Fig.2b) and are suppressed by the SAW. The monodispersity of drops with SAW is higher than without SAW. The reason for the oscillations is that after formation drops flow into a larger reservoir on the chip. The microfluidic channel from the location where drops are formed and the reservoir contains 8 drops. Hence, a correlation of every eighth drop would be expected as observed in the experiment. This unwanted correlation is reduced when using triggered SAW formation.

2.The decrease in the amplitude of the ACF we call "drift" occurs in both experiments, with and without SAW, and is of the same order. The drift is NOT caused or related to the SAW pinch of mechanism we want to demonstrate in this paper but present also, when the drop maker is run without SAW (Fig.2a). We assume that the slow drift observed for the drop maker is caused by a slow drift in the pumps. To substantiate this we modelled the different situations with and without SAW and determined the ACF. The results clearly show that the assumed model can explain and matches the experimentally observed ACF in both situations with and without SAW:

First, we model the drop ***formation without the SAW****. To account for the oscillations caused by the drops in the outlet channel we use a periodic function of the same periodicity of 8 drops : (40 *sin(n pi/4) , The drift we model by a linear drift n, with n running from 1 to 100 (one hundred droplets). The notation rnorm(100) means that finally we superpose a random noise to account for any non-periodic disturbance. The result is shown in Fig.1.*

*In a second simulation we just model linear drift superposed with random noise of the same level (30*rnorm) as shown in Fig.2. Finally, we model the situation* ***with SAW****. To get a good match with our experiments we have to lower the random noise level to 10*rnorm as shown in Fig. 3. This simulation with reduced noise and strongly suppressed oscillations reproduce the finding in Fig.2b in our manuscript quite well.*

Fig. 1: Linear drift superposed on a periodic oscillation and random noise:

f(n) = n + 40 * sin(n * pi/4) + 30 * rnorm(100), n=c(1:100)

Fig. 2: Linear drift and random noise:

f(n) = n + 30 * rnorm(100), n=c(1:100)

Fig. 3: Linear drift and reduced random noise (1/3 of original amplitude):

f(n) = n + 10 * rnorm(100), n=c(1:100)

rnorm: normal-distributed random numbers with standard deviation $\sigma = 1$

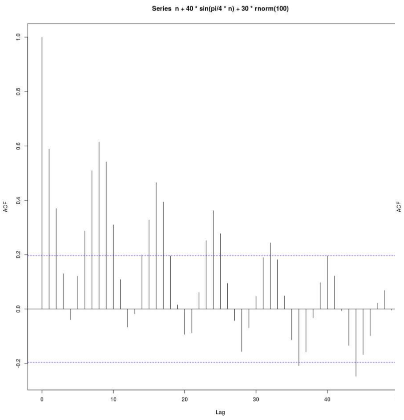

Fig. 1

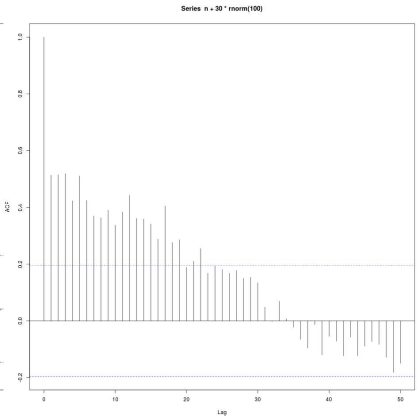

Fig. 2

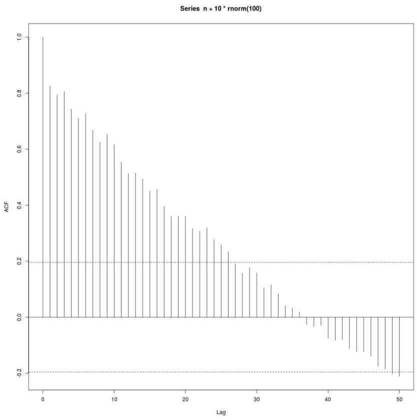

Fig. 3
